# Paediatric arterial ischemic stroke: acute management, recent advances and remaining issues

**DOI:** 10.1186/s13052-015-0174-y

**Published:** 2015-12-02

**Authors:** Margherita Rosa, Silvana De Lucia, Victoria Elisa Rinaldi, Julie Le Gal, Marie Desmarest, Claudio Veropalumbo, Silvia Romanello, Luigi Titomanlio

**Affiliations:** Department of Translational Medicine-Section of Pediatrics, Federico II University, Naples, Italy; Department of Paediatrics, Aldo Moro University of Bari, Bari, Italy; Department of Paediatrics, University of Perouse, Perugia, Italy; Paediatric Migraine & Neurovascular diseases Unit, Department of Paediatrics, Robert Debré Hospital, Paris Diderot University, Sorbonne Paris Cité, Paris, France; Paediatric Emergency Department, Robert Debré Hospital, Paris Diderot University, Sorbonne Paris Cité, Paris, France; Pediatric Emergency Department, INSERM U-1141 AP-HP Robert Debré University Hospital, 48, Bld Sérurier, 75019 Paris, France

**Keywords:** Paediatric stroke, Arterial ischemic stroke, Cerebral arteriopathy, Sickle cell disease, Moyamoya, Antithrombotic treatment, Thrombolysis

## Abstract

Stroke is a rare disease in childhood with an estimated incidence of 1-6/100.000. It has an increasingly recognised impact on child mortality along with its outcomes and effects on quality of life of patients and their families. Clinical presentation and risk factors of paediatric stroke are different to those of adults therefore it can be considered as an indipendent nosological entity. The relative rarity, the age-related peculiarities and the variety of manifested symptoms makes the diagnosis of paediatric stroke extremely difficult and often delayed. History and clinical examination should investigate underlying diseases or predisposing factors and should take into account the potential territoriality of neurological deficits and the spectrum of differential diagnosis of acute neurological accidents in childhood. Neuroimaging (in particular diffusion weighted magnetic resonance) is the keystone for diagnosis of paediatric stroke and other investigations might be considered according to the clinical condition. Despite substantial advances in paediatric stroke research and clinical care, many unanswered questions remain concerning both its acute treatment and its secondary prevention and rehabilitation so that treatment recommendations are mainly extrapolated from studies on adult population. We have tried to summarize the pathophysiological and clinical characteristics of arterial ischemic stroke in children and the most recent international guidelines and practical directions on how to recognise and manage it in paediatric emergency.

## Background

Stroke is defined as a sudden loss of brain function caused by a decreased cerebral blood flow. It can occur at all life stages but clinical presentation, pathophysiology and other clinical perspectives are variable depending on the patient’s age.

While many efforts and important clinical trials have been allocated to increase knowledge about adult stroke, paediatric stroke often remains an under-recognized nosological entity even among paediatricians, although it is an important cause of lifelong disability with a human and economic impact on families and on the society [1–3]. The estimated incidence of paediatric stroke is 1–6 per 100,000 children per year [4–6].

Rates of perinatal stroke (until 28 days after birth) are even higher, occurring in at least 1 in 3,500 live-births. Perinatal stroke differs from paediatric stroke in some clinical and pathophysiological aspects, mainly overlapping with global hypoxic ischemic encephalopathy and presenting with more aspecific and generalised signs such as apnea, hypotonia, poor feeding, seizures and irritability [7–11].

Differently, in children focal neurological deficits such as hemiplegia are the most frequently reported signs and the most frequently affected artery is middle cerebral artery (MCA).

In this review we have tried to focus on risk factors and underlying mechanisms, diagnosis, treatment, rehabilitation, knowledge translation and knowledge needs about paediatric arterial ischemic stroke (AIS).

## Risk factors for paediatric stroke

Presumptive risk factors for paediatric stroke differ in children compared with adults. Whereas adult risk factors are primarily related to arrhythmias, obstructive atherosclerotic arteriopathies and socioeconomic status, these are rarely found to be related to stroke in children [12, 13].

In several studies, such as the International Paediatric Stroke Study (IPSS), a wide range of underlying systemic factors were reported in the setting of childhood stroke, in particular: sickle cell disease (SCD), cardiac disorders, trauma, and major infections such as meningitis, sepsis and encephalitis. However, in the majority of the children, no underlying systemic disease was found [14].

**Fig. 1:**
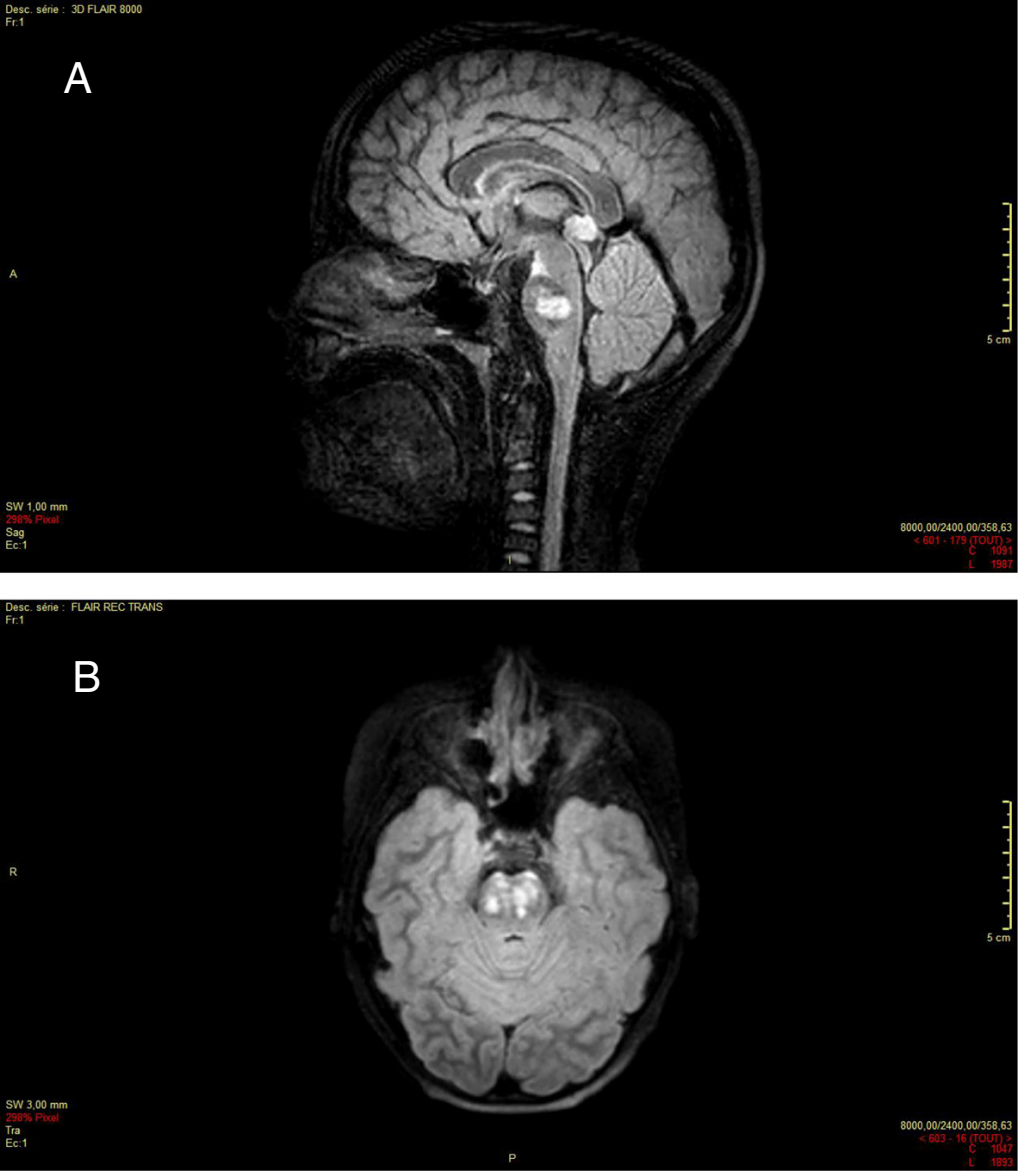
**a** Bilateral ischemic lesion of the ponto-mesencephalic junction, enhanced on the left side (day 1), 3d FLAIR, sagittal section. **b** Bilateral ischemic lesion of the ponto-mesencephalic junction, enhanced on the left side (day 1), axial section

**Fig. 2:**
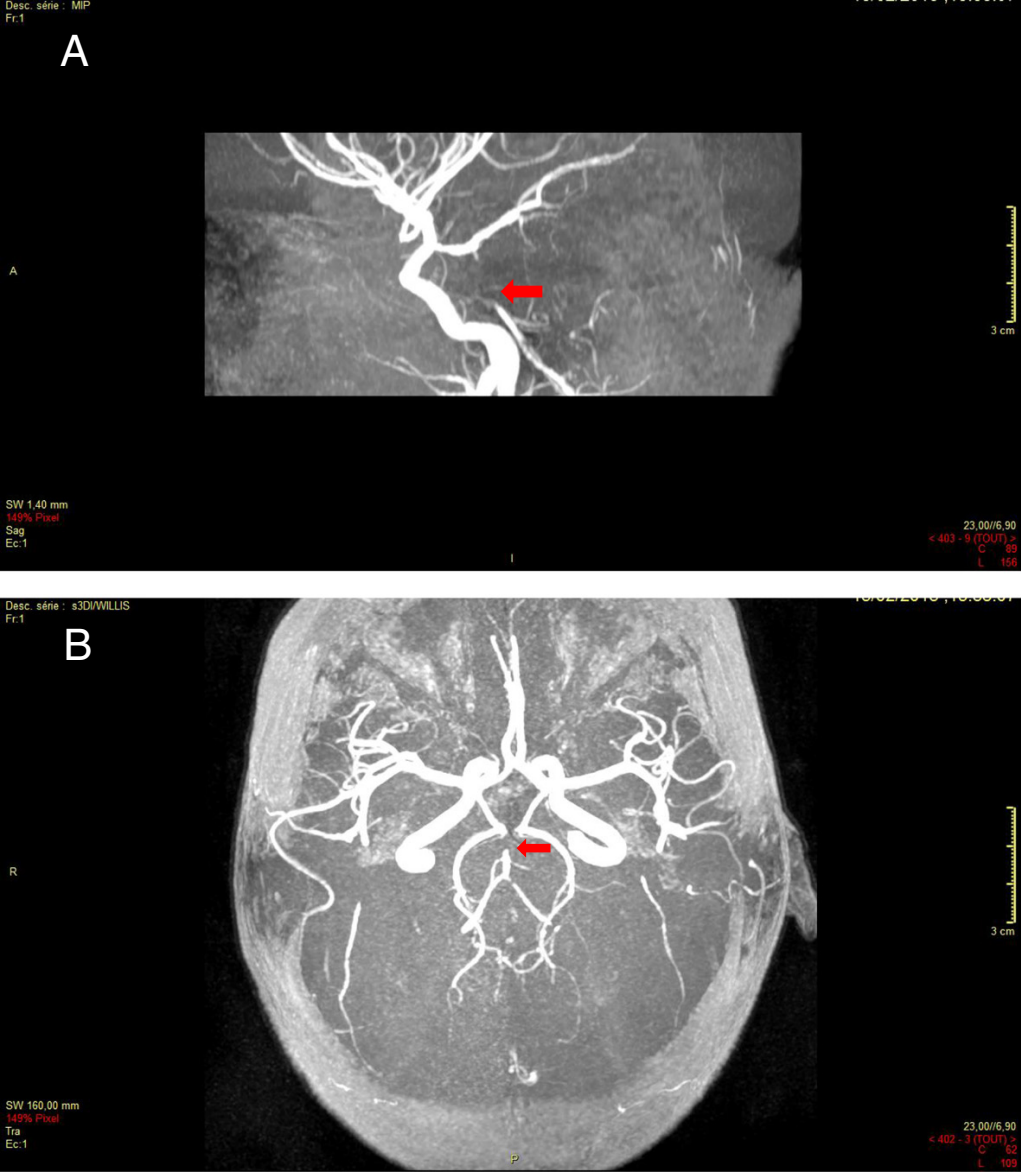
**a** Thrombosis of the superior portion of the basilar trunk, acute phase (day 0), 3 DI Willis, sagittal section. **b** Thrombosis of the superior portion of the basilar trunk, acute phase (day 0), 3 DI Willis, axial section

**Fig. 3:**
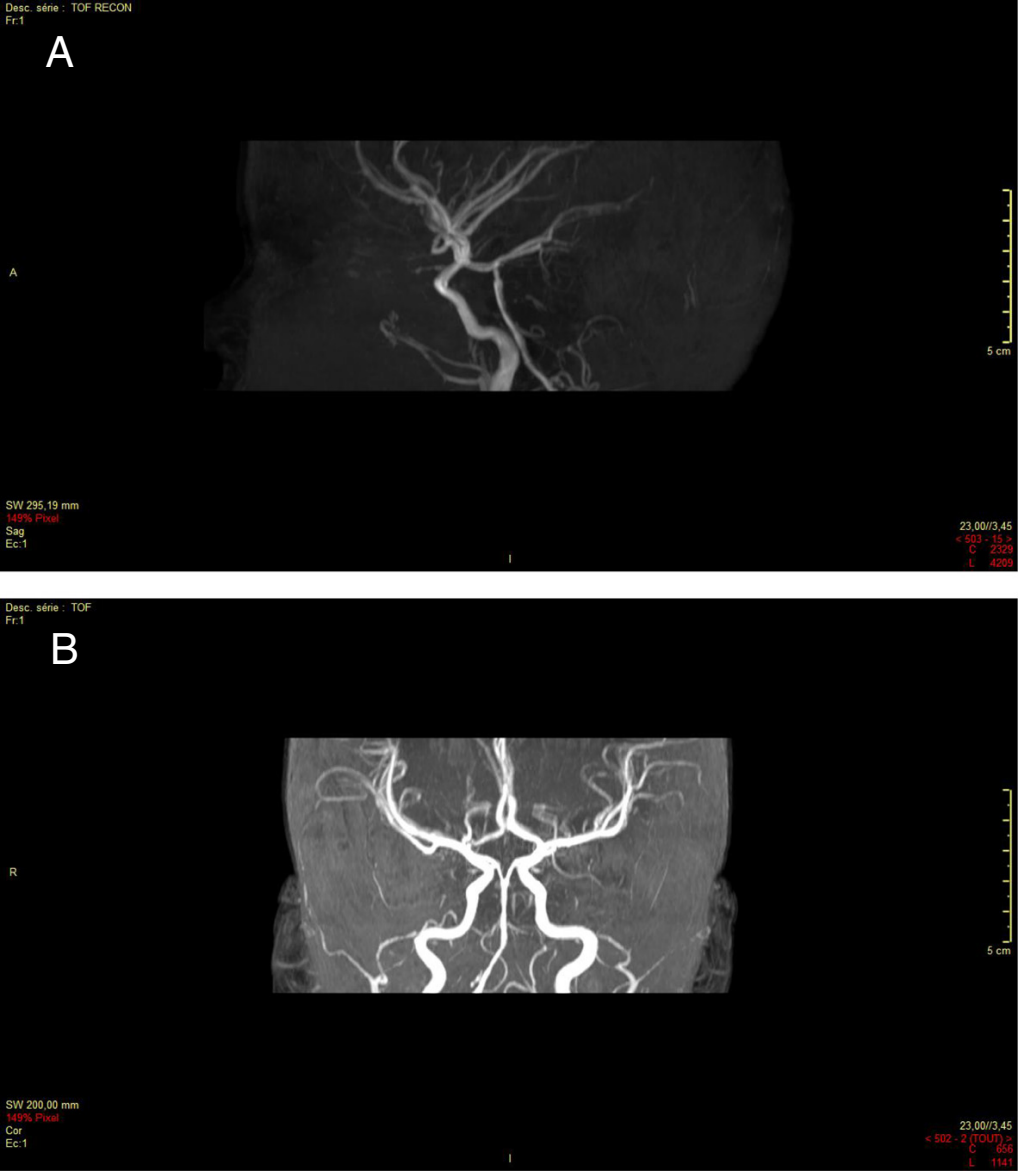
**a** After thrombectomy of the basilar trunk (day 1), 3DI Willis, sagittal section (before thrombectomy: see Fig. 2a). **b** After thrombectomy of the basilar trunk (day 1), 3DI Willis, axial section (before thrombectomy: see Fig. 2b)

### Arteriopathy

With the advances in neuroimaging, arteriopathy appears to be the predominant underlying mechanism, causing 53 % of paediatric strokes. Furthermore, it represents the greatest predictor of recurrence, emphasising its role as a treatment target for secondary stroke prevention [13, 15].

The most common established arteriopathy in paediatric stroke is an acquired unilateral intracranial arteriopathy associated with basal ganglia stroke, characteristically involving the junction of the distal internal carotid artery, the proximal MCA and the proximal anterior cerebral artery (ACA). This condition was originally designed as a transient cerebral arteriopathy (TCA) and characterised by its time course, by an unilateral location, and by the absence of a long-term progression [16, 17]. On initial traditional imaging, TCA may be not distinguishable from a progressive arteriopathy such as Moyamoya disease (MMD) or from a vasculitis that presents unilaterally, with a different course and prognosis. Few clinical and radiological parameters may help predict the course of childhood unilateral intracranial arteriopathy. It has been described that patients with progressive arteriopathy more often show arterial occlusion, ACA involvement and abnormal collateral vessels and that a mainly cortical localization is associated with poor functional outcome. The distinction between TCA and progressive arteriopathy may require further radiological assessment, such as magnetic resonance angiography (MRA) and conventional angiography, and, in general, its worsening after 6 months or a bilateral involvement suggests an arteriopathy other than TCA [16, 18].

Conscious of the limitations in classifying arteriopathies on initial imaging, the IPSS has recently suggested the term “focal cerebral arteriopathy” of childhood (FCA) to describe children with an unilateral arterial stenosis without an apparent underlying cause at presentation. These arteriopathies are clinically important because their presence indicates an increased risk for recurrent stroke and vascular imaging of intracranial and neck vessels are imperative to detect them.

The underlying mechanisms for these unilateral, monophasic arteriopathies are poorly understood. Investigators originally proposed that the basis for this disorder was inflammatory, but currently the possible role of infection is the most plausible hypothesis. In particular, chickenpox is associated to AIS with an absolute risk of 1 in 15,000 children and varicella-associated AIS includes nearly one third of childhood AIS. It has been reported that in young children with AIS there is a 3-fold increase in preceding varicella infection compared with population rates and this prevalence was higher than the annual prevalence of chickenpox reported in the regional population [19]. The fact that post-varicella angiopathy, consisting in a non-progressive arteriopathy, has similar arterial imaging but a presumed infectious cause suggests that TCA might sometimes have a link to infectious mechanisms [17]. Furthermore, acute infection has been shown to be a risk factor and a potential trigger for spontaneous cervical artery dissection and upper respiratory infections (URI) have recently been related to transient arteriopathy. In the IPSS, a recent URI was noted in 9.1 % of children with a cerebral arteriopathy and based on the preliminary results of the Vascular Effects of Infection in Pediatric Stroke (VIPS) study, infections ≤1 week prior to stroke/interview date conferred a 6.5-fold risk of AIS and the most common type of infection described was upper respiratory. Also, children reported to have had some/few/no routine vaccinations were at higher risk of AIS than those receiving all or most, suggesting that a higher frequency of upper respiratory diseases may increase the incidence of pediatric AIS and that their prevention might prevent stroke in pediatric patients [20–23]. In conclusion, although many underlying diseases have been reported in the setting of childhood AIS, emerging research demonstrates that non-atherosclerotic intracerebral arteriopathies in otherwise healthy children are prevalent. In particular, these conditions are related to parainfectious aetiology, inflammatory reaction and altered inflammation signalling [13].

### Genetical factors and Moyamoya syndrome

Genetically determined arteriopathies are increasingly recognised as a cause of childhood AIS. The list continues to expand, including *COL4A1, ACTA2*, and pericentrin (*MOPD2*) mutations, and syndromes such as Alagille’s and PHACE.

The most studied syndrome is the Moyamoya disease which accounts for 6 % of childhood AIS. It is characterized by a usually bilateral progressive stenosis or occlusion of the intra-cranial internal carotid apices, involving the anterior and medial cerebral arteries. MMD is connected to the *RNF213* gene in a substantial portion of Japanese patients and other mutations of *BRCC3/ MTCP1* and *GUCY1A3* have recently been reported in more complex Moyamoya syndromes [24].

In 2014, a genetic arteriopathy caused by a deficiency of adenosine deaminase 2 (ADA2) was reported with clinical features that included intermittent fever, lacunar strokes beginning in early childhood and livedoid rash; histopathological changes included compromised endothelial integrity, endothelial cellular activation and inflammation [16, 25].

### Hereditary coagulopathies and trombophilia

One or more prothrombotic states have been identified in 20 to 50 % of children presenting with AIS [26]. The main mutations associated with prothrombotic states are described in factor V Leiden, prothrombin G20210A, methylenetetrahydrofolate reductase (MTHFR; C677 T and A1298 C), protein C, protein S, antithrombin and lipoprotein (a) [27–29]. Most paediatric stroke experts believe that coagulopathy represents a potential risk factor for stroke usually working in combination with other factors, rather than being an independent causative mechanism [20, 30–33]. Thus, it is reasonable to search for the more common prothrombotic states in patients with another identified stroke risk factor and in patients with a history of ischemic or thrombotic stroke (in this case, oral contraceptives may be discontinued in adolescents). If homocysteine is found to be elevated, aspecific diet or supplementation with folate, vitamin B6, or vitamin B12 might be considered and, in general, patients who are found to have a prothrombotic tendency should be referred to a haematologist [13, 34, 35].

### Sickle cell disease

Children with SCD represent another important group of patients with a high risk of arteriopathies and stroke. Prior to modern primary prevention strategies, up to 11 % of children with SCD had a clinical stroke by the age of 20 years. In 1992, transcranial Doppler (TCD) was found to be effective in identifying SCD patients at a high risk for stroke and currently primary prevention is possible through chronic red blood cell transfusions in SCD patients with elevated TCD velocity. This approach has decreased the prevalence of overt stroke approximately to 1 % [13, 36–38].

### Metabolic diseases

Metabolic infarctions are a rare but important condition for the paediatric population. Energy depletion leads to ischemic lesions in mitochondrial disorders. In urea cycle disorders (especially OTCD), toxic deposits lead to the destruction of cerebral tissue. For this reason, metabolic infarctions do not occur in a specific vascular territory (MELAS for example shows a predilection for occipital infarctions). Other metabolic problems such as Fabry disease lead to a focal arteriopathy [15, 39].

### Congenital and acquired heart disease

Heart disease is still recognized as a presumed risk factor for paediatric stroke showing a high frequency in case series (19 % of children with arterial thrombosis according to the Canadian Paediatric Ischemic Stroke Registry) [40]. Complex congenital heart lesions with right-to-left shunting and cyanosis are particularly prone to cause stroke (in particular if uncorrected or in the peri-operative period), but stroke has also been described in children with other cardiac congenital lesions and occasionally with acquired disorders of the myocardium or cardiac valves [12, 13, 34]. (For a summary of paediatric stroke risk factors: see Table 1).

**Table 1:** Risk factors for pediatric AIS

Artheriopathies	Arterial fibromuscular dysplasia, arteriovenous malformation, arterial dissection, Moyamoya disease, transient cerebral arteriopathy of childhood, primary central nervous system vasculitis, cranial radiotherapy
Vasculitis	Meningitis, postinfectious systemic lupus erythematosus, polyarteritis nodosa, granulomatous angiitis, Takayasu’s arteritis, rheumatoid arthritis, dermatomyositis, inflammatory bowel disease, hemolytic-uremic syndrome, drug abuse
Hematologic disorders and coagulopathies	Hemoglobinopathies (sickle cell anemia, sickle cell-hemoglobin C, sickle-thalassemia), purpura, thrombocytosis, polycythemia, disseminated intravascular coagulation, leukemia or other neoplasms, congenital coagulation defects, oral contraceptive use, liver dysfunction with coagulation defect, vitamin K deficiency, Lupus anticoagulant, anticardiolipin antibodies
Metabolic disorders	Mitochondrial disorders (MELAS syndrome), urea matabolic disorders, homocystinuria, aminoaciduria, glutaric acidemia type I, lysosomal disorders, Fabry’s disease
Heart diseases	Congenital malformations (ventricular/atrial septal defect, patent ductus arteriosus, aortic/mitral stenosis, coarctation, complex congenital heart defects);
	Acquired (Rheumatic heart disease, endocarditis, myocarditis, arrhythmia)
Traumatic	Child abuse, post-traumatic arterial dissection, blunt cervical arterial trauma, arteriography, post-traumatic carotid cavernous fistula, penetrating intracranial trauma

## Diagnosis of paediatric stroke

Diagnosis of paediatric stroke is not simple and requires a neuroimaging reference that at present is considered essential to confirm the neurovascular origin of symptoms. Many cases receive delayed or *post-mortem* diagnosis, given the complexity of the presentation and the potential ambiguity of the clinical findings. A median time of 25 h between the onset of symptoms and the radiological evidence of stroke has been observed and only one third of the affected children are diagnosed within a delay of six hours [41–43].

### Signs and symptoms

Clinical presentation of paediatric stroke is variable depending on the patient’s age and the involved artery.

We have already explained above that perinatal stroke usually presents with more subtle and aspecific signs; more generally, in younger children, stroke symptoms are usually aspecific (seizures and altered mental status may be the only manifestation of clinical stroke, in particular if age < 1 year) while in older children they become more specific, often presenting with focal neurological deficits such as hemiplegia.

In Table 2 we have summarized the clinical presentation of paediatric stroke according to the involved cerebral territory. In general, the presence of hemiparesis, disphasia/aphasia (not limited to stroke of the dominant side in childhood, because of immature lateralization of language), and hemianopsia suggests a suvratentorial cerebral territory involvement, while ataxia is a typical symptom of an infratentorial stroke, but not limited to cerebellar lesions [44]. Headaches are present in 30 % of children and considerable differential diagnosis are hemiplegic migraine or dissection of carotid or vertebral artery [20].

**Table 2:** Clinical presentation of stroke depending on the involved artery

Vascular territory	Symptoms
Internal carotid artery	Hemiparesis, hemianopsia, aphasia
Anterior cerebral artery	Hemiparesis (legs+++)
Middle cerebral artery	Hemiparesis (arms+++), hemianopsia, aphasia
Posterior cerebral artery	Hemiparesis, hemianopsia, ataxia
Basilar artery	Sensory disturbance, nystagmus, ataxia, breath alterations
Cerebellar artery	Sensory disturbance, nystagmus, ataxia, tremor, dysarthria, vertigo, vomiting

Seizures have been described in 20–48 % of cases and may be part of paediatric stroke clinical presentation in all ages and independently from stroke subtype [17]. If they occur in the first 24 h of stroke onset, a higher risk for developing of Epilepsy in the next 6 months has been observed [45].

### Clinical history and physical examination

When collecting the clinical history, the physician should consider risk factors for paediatric stroke (Table 1), investigating ethnic origin, familial history and in particular a positive family history for coagulopathies, and cerebrovascular, metabolic or immunologic diseases. Particular attention should be given in the personal history to the presence of sickle cell disease or congenital heart disease, to a history of head or neck trauma, to unexplained fever and recent infections (especially chickenpox), vasculitis, drug ingestion and blood disorders.

A complete physical and neurological examination should be performed, considering the potential correspondence between neurological deficit and specific brain territory involved (Table 2). Signs of systemic diseases or cardiovascular diseases that increase the risk of stroke and skin lesions indicating trauma or infections should be looked for.

### Neuroimaging

In front of a child with acute neurological alterations with the prevision of a thrombolytic or neurovascular intervention, Magnetic Resonance Imaging (MRI) or non-contrast Computer Tomography (CT) should be performed.

In particular, non-contrast CT is easily available in emergency and can adequately exclude haemorrhagic stroke (a contraindication to thrombolytic therapy) or a mass effect produced by parenchymal abnormalities, furthermore it may identify cerebral venous sinus thrombosis and low-density lesions in arterial ischemic stroke (for example, hypoattenuation of the lentiform nucleus in acute ischemia of the lenticulostriate territory or insular ribbon sign in cytotoxic edema of the insular cortex). However, CT is usually normal within the first 12 h after the onset of symptoms [46, 47].

MRI with diffusion-weighted imaging remains the “gold standard” for the investigation of arterial ischemic stroke in infants and children, due to its greater sensitivity and specificity [47, 48], but it is rarely available in the the hyperacute phase managed in emergency departments. Typical MRI findings in patients with acute cerebral ischemia include hyperintense signal in white matter on T2- weighted images and fluid-attenuated inversion recovery images, with a resultant loss of grey matter–white matter differentiation. Other findings may be sulcal effacement and mass effect, loss of the arterial flow voids and stasis of contrast material within vessels in the affected territories [49–51] (Fig. 1)*.* Otherwise, in paediatric patients < 2 years, the incomplete myelinisation compromises the efficacy of fluid-attenuated inversion recovery MRI sequences [52]. In patients with SCD or other predisposing diseases, MR images may be characterised by a coexistence/overlapping of the acute cerebrovascular injury and chronic sequelae of previous AISs. The diagnostic efficiency of MRI can be further improved by perfusion techniques to differentiate between infarct core and penumbra and by Susceptibility-Weighted Imaging to estimate infarct progression [53, 54]. Magnetic Resonance Angiography allows the identification of the involved vascular territory, and can be useful to evaluate the effectiveness of thrombectomy (Figs. 2 and 3)*.* Vascular imaging of the carotid and vertebral arteries by duplex ultrasonography, CT angiography (CTA), MRA or catheter angiography should be considered within 24 h of the stroke [55, 56].

### Neuroimaging in Moyamoya disease

Conventional angiography or MRA are required to identify Moyamoya disease [34, 57]. In particular, MRI-MRA may be sufficient to diagnose MMD when they find the typical pattern of stenosis or occlusion of the terminal part of the internal carotid artery and/or the proximal portion of the ACA and/or MCA, the abnormal arterial vascular network near the steno-occlusive lesions with the appearance of a “puff of smoke” or two or more flow voids in the basal ganglia, the sparing of posterior circulation. The diagnosis is “definite MMD” if these findings are bilateral and is “probable MMD” if they are unilateral. The “Ivy sign” may also be found from FLAIR sequences, consisting in linear hypersignals that follow a sulcal pattern and indicating a slowed cortical blood flow [58, 59].

Cerebral conventional angiography remains the gold standard for diagnosis and classification of MMD and is more reliable when applied to smaller vessel occlusions; however, because of its invasivity, conventional angiography is realized in limited circumstances, such as uncertain diagnosis or progression of the angiopathy during follow-up, pre-surgical management, evaluation after surgical revascularization. The Suzuky classification, based on the physiological reorganization process called “*internal carotid -external carotid conversion*”, remains the reference standard for evaluating disease severity [60].

The progressive bilateralization and the decrease of global cerebral blood flow with a predominant posterior distribution are more frequent in pediatric patients with MMD than in adult patients. Assessment of cerebral perfusion in MMD may be obtained through cerebral hemodynamic techniques (single photon emission computed tomography [SPECT], perfusion CT, xenon-enhanced CT and MRI-based dynamic susceptibility contrast and arterial spin labeling) and metabolic techniques (positron emission tomography [PET]-scan) [61–63].

### Optional investigations

Laboratory examinations in the acute setting, including routine chemistry, electrolytes, hematology and coagulation should be conducted as part of the initial evaluation [55]. Other investigations can be useful for differential diagnosis with meningo-encephalitis (generally associated with fever, headache and altered consciousness), metabolic disorders, epilepsy (acute hemiplegia may be a sign of post-seizure Todd-Palsy), idiopathic intracranial hypertension, empyema and intracranial abscess, acute disseminated leukoencephalitis, cerebellitis, tumor, etc. (Table 3: Differential diagnosis of paediatric stroke) and could be evaluated depending on the specific clinical condition [64].

**Table 3:** Differential diagnosis of ischemic stroke

Hemorrhagic stroke	
Cerebral venous sinus thrombosis	
Hemiplegic migraine	
Todd’s Palsy	
Intracranial infections (meningitis, brain abscess, herpes simplex encephalitis)	
Cerebellitis	
Alternating hemiplegia	
Metabolic disorders (MELAS)	
Tumors	
Acute disseminated leukoencephalitis	
Reversible posterior leukoencephalopathy syndrome	
Idiopathic intracranial hypertension	
Drug toxicity	
Psychogenic diseases	

### After-diagnosis investigations

Several studies may be helpful after the diagnosis of paediatric stroke for the on-going evaluation and management of the patient.

Electrocardiogram and transthoracic or transesophageal echocardiogram are mandatory for a cardiological work-up both in patients with known congenital heart malformations or with suspicion of cardiac disease and in patients with stroke of undetermined aetiology. According to the Royal College of Physicians Guidelines (RCP; 2004), transthoracic cardiac echocardiography should be undertaken within 48 h of presentation with AIS [35].

Haemoglobin electrophoresis may be indicated to identify hemoglobinopathies which represents a risk factor for stroke (SCD, sickle cell-hemoglobin C, sickle-thalassemia).

A full evaluation for thrombophilia is reasonable in all children with stroke and may include protein C and protein S deficiency, antithrombin III, heparin cofactor II, plasminogen, vonWillebrand antigen, factor VIII, factor XII, factor V Leiden, activated protein C resistance, prothrombin 20210 gene, serum homocysteine, MTHFR, lipoprotein (a), and antiphospholipid antibodies.

Screening for metabolic, immunologic disorders and infections related to stroke (in particular, VZV, immunodeficiency syndrome, meningovascular syphilis, infective endocarditis) and research of drug intoxication (especially sympathomimetics) could be evaluated depending on the specific clinical condition [34, 65, 66].

## Treatment of pediatric stroke

The main target of treatment of paediatric stroke is the protection of the developing brain by minimizing acute brain injury, preventing neurodevelopmental impairment and disability.

Because of the lack of data from paediatric studies, there is no evidence-based treatment for paediatric stroke. Nevertheless recent guidelines such as the Canadian Best Practice Guidelines (CBP; 2010), the American College of Chest Physicians Evidence-Based Clinical Practice Guidelines (CHEST; 2012), the American Heart Association Guidelines (AHA; 2008) and the RCP 2004, that extrapolate data both from an adult and a paediatric population, may help the clinician [35, 55, 67].

Firstly, children with acute stroke may be hospitalized in a clinical unit with the possibility of continuous monitoring. In selected cases transfer to intensive care units is needed [68, 69].

### Acute phase treatment

In general, guidelines consistently recommend antithrombotic treatment for paediatric AIS; before starting the treatment, exclusion of haemorrhagic stroke is mandatory.

The specific indications of anticoagulant therapy remain controversial. Most guidelines recommend treatment with *Unfractioned heparin (UFH) or low molecular-weight heparin (LMWH)* in children with a proven arterial dissection or cardioembolic stroke or during the diagnostic evaluation period, until cardioembolism or an arterial dissection have been excluded. Nevertheless, the RCP recommends initial treatment of childhood AIS with aspirin 5 mg/kg until an indication for anticoagulation is found and, according to CBP 2010, the use of anticoagulation in children with cardiac embolism stays controversial and the risk of haemorrhagic transformation of the infarct must be considered and individualised with the help of paediatric cardiologists and neurologists.

Heparin may be considered taking into account a risk/benefit balance because of the reported heparin-induced thrombocytopenia and thrombotic complications. According to guidelines, LMWH can be started safely in paediatric AIS at a dose of 1 mg/kg twice a day. Heparin-based anticoagulation, when employed, is best monitored with anti-factor Xa activity considering as a therapeutic range 0.35 to 0.7 anti-factor Xa activity U/mL for unfractionated heparin and 0.5 to1.0 U/mL for LMWH in a sample taken 4 to 6 h after subcutaneous injection.

### Pharmacological and mechanical thrombolysis

Guidelines do not recommend the use of both thrombolysis by tissue plasminogen activator (t-PA, Alteplase) and mechanical thrombectomy in children outside specific research protocols.

Despite the lack of evidence of safety and efficacy in paediatric patients, these techniques have been applied to children with AIS based on evidence from a few case reports and case series, as well as extrapolation from the adult literature. In particular, 34 published cases of paediatric endovascular treatment including intra-arterial alteplase were reviewed in 2011 and about 2 % of children in the US with AIS are treated with intravenous thrombolysis [70–72].

Adult AIS patients are now routinely given *systemic tPA* when presenting within 3 h from stroke onset, at a usual dose of 0.9 (max 90 mg) mg/kg, with a 10 % of the dose as a bolus and the remaining as a 1-h infusion [73]. Concerning paediatric stroke population, a study was started to define intravenous alteplase safety and dose-finding within 4–5 h from stroke onset, but it was halted because of insufficient recruitment [74, 75].

*Intra-arterial thrombolysis*, which extends the window of treatment by endovascular approaches from 3 to 6 h,, is successfully utilized in the adult population. In paediatric patients no randomised controlled trials are available but small cohorts underwent the procedure with encouraging results, excepted for one patient who died for MCA occlusion, while suffering from carotid bifurcation occlusion (which is known to present a poor prognosis in adults).

*Endovascular mechanical thrombectomy* has also been used in children despite, as already explained, guidelines advise against its exportation in the paediatric population. The potential candidates for this approach may be paediatric patients with a large intracranial artery occlusion. Safety guidelines are needed considering the risks associated with the treatment of children as small adults and the inappropriateness of devices often designed for adult use. Concerning decision making, the entity of the neurological deficits, the dimension of the involved artery, the MRI evidence of preservation of brain tissue and the familiarity of the centre with paediatric stroke treatments, may be considered [71, 76].

### Secondary prevention

Even in the absence of randomized clinical trials for the acute treatment of AIS in children, most experts agree that *aspirin* use is reasonable for secondary stroke prevention in children that are not at high risk of recurrent embolism and that are not affected by a hypercoaguable disorder. The dose varies from 1–5 mg/kg/die following the principal guidelines (a dose of 3 to 5 mg/kg per day is reasonable considering a reduction to 1 to 3 mg/kg in the case of dose-related side effects, CHEST 2012). The optimum duration of aspirin therapy is poorly defined, although a minimum of 2 years of therapy is suggested. The increased risk of Reye’s syndrome should be considered. If children are unable to take aspirin, clopidogrel may be considered as an alternative treatment at dosages of about 1 mg/kg per day, while the combination of clopidogrel and aspirin should be used with caution [77].

*LMWH or warfarin* are recommended in secondary prophylaxis by the RCP guidelines in children with cardioembolism or extracranial arterial dissection and may be considered in children with cerebral venous sinus thrombosis according to CBP 2010 and in children with recurrent AIS despite aspirin treatment according to CHEST 2012 and CBP 2010. The AHA guidelines also suggest to consider anticoagulation in children with antiphospholipid antibody syndrome.

Surgical intervention may be considered for AIS secondary to cardioembolic causes in the case of demonstrated right-to-left shunts (eg, patent foramen ovale) [67].

Some institutions have recently successfully developed a paediatric service for anticoagulation safety [78] and a large recent nonrandomized study found no difference in secondary stroke prevention with the use of warfarin versus aspirin after cervical artery dissection [79].

In conclusion, the comparison between antiplatelet and anticoagulant drugs for safety and efficacy remains controversial. Although preliminary studies suggest that anticoagulation may be safe in children with AIS (even in those with arteriopathy), the need of a randomised clinical trial to establish the optimum antithrombotic treatment of paediatric stroke and its secondary prevention persists.

### Treatment in particular conditions

In SCD primary management consists of evaluation by a multi-disciplinary team (a hematologist, neurologist, neuroradiologist and a transfusion medicine specialist); prompt neuro-imaging, and an initial simple blood transfusion followed immediately by an exchange transfusion or only exchange transfusion (with the aim of exchanging hemoglobin A with sickle cells hemoglobin without increasing cerebral blood volume) if the hemoglobin is > 4 gm/dL and < 10 gm/dL. Standard therapy for secondary prevention of stroke and silent cerebral infarcts includes regular blood transfusion therapy and in selected cases, hematopoietic stem cell transplantation [37, 38, 76].

In metabolic diseases, the first-line therapy is correction of the underlying metabolic defect. In Moyamoya, therapy is mainly surgical revascularization to establish an alternative source of vascular flow for hypoperfused territories. The existence of different patterns of Moyamoya disease and the rarity of this condition, solicit an individualized decision making and the patient should be referred for evaluation to a centre with expertise in evaluating patients for surgical revascularisation [34]. Despite a vast literature about Moyamoya, the need of controlled clinical trials to guide the decision of therapy persists [24, 80, 81].

### Supportive care

Patients should receive supportive care with particular attention to optimisation of glycaemia, volemia, oxygenation and blood pressure and to prevent and correct hyperthermia and infections [34, 82]. Management of physiologic clinical and laboratory status may follow the general statements for the acutely ill child [83].

To prevent secondary brain injury, seizures may be controlled (with eventual continuous electroencephalographic monitoring and antiepileptic medications in the case of clinical or electrographic seizures) and a normal oxygenation may be assured. No benefits have been observed with oxygen supplementation in the absence of hypoxemia neither with the use of hypothermia in paediatric stroke [34, 84, 85].

Concerning blood pressure control, management of elevated blood pressure in paediatric patients with AIS remains controversial due to the lack of evidence for a particular pressure range target. In general, extreme blood pressure elevation (e.g. systolic > 220 or diastolic > 120 mmHg) may be treated to reduce the blood pressure by ~15 %, and not more than 25 %, over the first 24 h with a gradual reduction thereafter. Excessive lowering of blood pressure should be avoided as this may exacerbate or may induce ischemia [57, 72].

Artificial hyperventilation and osmotic agents may be considered in children with intracranial hypertension. In the case of necessary hydrocephalus drainage or decompressive craniectomy for massive cerebellar infarction, transfer to a neurosurgery department must be evaluated.

### Rehabilitation

Given the plasticity of the young brain, rehabilitation for children following stroke can likely lead to vast improvements in long-term outcomes, with a favourable impact in long-term morbidity, quality of life and emotional health for the child and the family [86]. A multidisciplinary team should be involved in paediatric stroke rehabilitation utilizing a biopsychosocial model and taking into account that the emotional well-being of the family following a stroke may influence the recovery of the child [57, 73]. Adjunctive therapies have shown encouraging results in animal models and are now being studied in both adults and children with stroke, including constraint-induced movement therapy (CIMT), bimanual training and transcranial magnetic stimulation. These techniques have been defined as relatively safe, with a maximum benefit when performed in crucial periods of early development and with hopeful improvements in motor long-term follow-up [87–99].

## Conclusions

Paediatric stroke is a relatively rare disease associated to a significant morbidity and mortality so to deserve the attention of clinicians. Stroke may have a subtle presentation in particular among new-borns and in younger children, but in general it should be taken into account in the differential diagnosis of any child presenting with new-onset focal deficits, altered speech, ataxia, important headache, seizures or alterations of mental state. Advanced imaging devices have improved the diagnosis of paediatric stroke; MRI techniques are considered the “gold standard” and different combinations of radiological exams may be applied according to current guidelines and to the specific clinical condition. Although recently published guidelines, often extrapolating data from an adult population, may help the clinician in paediatric stroke treatment decisions, controlled randomised studies to determine the optimal acute treatment of paediatric stroke and its secondary optimal prevention and rehabilitation are critically needed.

## Abbreviations

ADA2: Adenosine Deaminase 2
AHA 2008: American Heart Association Guidelines 2008
AIS: Arterial Ischemic Stroke
CBP 2010: Canadian Best Practice Guidelines 2010
CHEST 2012: American College of Chest Physicians Evidence-Based Clinical Practice Guidelines 2012
CIMT: Constraint-Induced Movement Therapy
CT: Computed Tomography
CTA: Computed Tomography Angiography
FCA: Focal Cerebral Arteriopathy
FLAIR: Fluid Attenuated Inversion Recovery
GSC: Glasgow Coma Scale
IPSS: International Paediatric Stroke Study
LMWH: Low Molecular-Weight Heparin
MCA: Middle Cerebral Artery
MELAS: Myopathy, Encephalopathy, Lactic Acidosis, and Stroke-like episodes
MMD: Moyamoya disease
MTHFR: Methylenetetrahydrofolate Reductase
MR: Magnetic Resonance
MRA: Magnetic Resonance Angiography
MRI: Magnetic Resonance Imaging
OTCD: Ornithine Transcarbamylase Deficiency
PET: Positron Emission Tomography
RCP 2004: Royal College of Physicians Guidelines 2004
SCD: Sickle Cell Disease
SPECT: Single Photon Emission Computed Tomography
tPA: tissue Plasminogen Activator
TCA: Transient Cerebral Arteriopathy
TCD: Transcranial Doppler
UFH: Unfractioned Heparin
URI: Upper Respiratory Infection
VIPS: Vascular Effects of Infection in Pediatric Stroke
